# Genetic and Genomic Pathways of Melanoma Development, Invasion and Metastasis

**DOI:** 10.3390/genes12101543

**Published:** 2021-09-28

**Authors:** Jyoti Motwani, Michael R. Eccles

**Affiliations:** 1 Department of Pathology, Dunedin School of Medicine, University of Otago, Dunedin 9016, New Zealand; mail.jyotim@gmail.com; 2 Maurice Wilkins Centre for Molecular Biodiscovery, Auckland 1010, New Zealand

**Keywords:** melanoma, mutations, transcriptome, epigenome, clonal evolution, cancer stem cells, phenotype switching

## Abstract

Melanoma is a serious form of skin cancer that accounts for 80% of skin cancer deaths. Recent studies have suggested that melanoma invasiveness is attributed to phenotype switching, which is a reversible type of cell behaviour with similarities to epithelial to mesenchymal transition. Phenotype switching in melanoma is reported to be independent of genetic alterations, whereas changes in gene transcription, and epigenetic alterations have been associated with invasiveness in melanoma cell lines. Here, we review mutational, transcriptional, and epigenomic alterations that contribute to tumour heterogeneity in melanoma, and their potential to drive melanoma invasion and metastasis. We also discuss three models that are hypothesized to contribute towards aspects of tumour heterogeneity and tumour progression in melanoma, namely the clonal evolution model, the cancer stem cell model, and the phenotype switching model. We discuss the merits and disadvantages of each model in explaining tumour heterogeneity in melanoma, as a precursor to invasion and metastasis.

## 1. Introduction

Melanoma is a life-threatening form of skin cancer that is derived from malignant transformation of the melanocyte cell lineage. It accounts for 80% of all the skin cancer deaths [1], as well as 85% of eye cancers [2]. A recent study, which was conducted on the populations of six different countries, using three decades of cancer registry data, demonstrated that Australia and New Zealand have the highest rates of melanoma in the world, with as high as 50 cases of melanoma per 100,000 people in parts of Queensland and North Island of New Zealand [3]. Early stage melanoma, when it is still a thin primary, can be easily cured by surgical resection. However, there are fewer treatment options available for patients with metastatic melanoma. Although, advanced targeted therapies, immunotherapies, and a combination of targeted and immunotherapies have improved the treatment and survival rate of melanoma patients, innate and acquired resistance involving metastatic melanoma lesions, as well as toxicity in response to these treatments, are still major problems with these therapies [4,5,6,7]. One of the primary factors underlying resistance to drug therapies is the heterogeneous nature of melanoma [8,9]. Tumour heterogeneity is believed to be a major determinant of invasion and metastasis. Yet, the mechanisms leading to tumour heterogeneity are incompletely understood, and are linked to several contemporary theories of melanoma progression, namely, the theory of clonal evolution with somatic mutation, the cancer stem cell origin of heterogeneity theory, and the phenotype-switching reversible behaviour theory, which we discuss in this review together with concepts behind these theories, and potential roles that genetic and genomic alterations play in the progression of primary melanoma to invasion and metastasis.

## 2. Mutational Landscapes of Melanoma

### 2.1. The Mutational Landscape of Somatic Melanomas

Melanoma has among the highest rate of somatic mutations of any cancer type [10]. Somatic mutations are more frequent in melanomas than germline mutations. Mutations in the v-Raf murine sarcoma viral oncogene homolog B proto-oncogene (*BRAF*) are the most common coding region mutations that occur in 33–65% of cutaneous malignant melanomas [11]. The most recurrent mutation, BRAF^V600E^, which involves a valine to glutamate point mutation at residue 600, hyper-activates the mitogen-activated protein kinase (MAPK) pathway and is involved in cell proliferation [12]. This mutation is more frequently identified in melanocyte nevi, indicating that it is associated with early pre-neoplastic stages of melanoma progression [13]. Other *BRAF* mutations such as BRAF^V600K^ (7–20%), BRAF^V600D^ and BRAF^V600R^ are less common [14]. *BRAF* mutations have been identified in melanomas as a result of intermittent sun exposure, whereas melanomas arising from skin that was unexposed to the sun rarely showed *BRAF* mutations [15]. This observation indicates that intermittent sun exposure plays a causative role in inducing *BRAF* mutations. Given the frequent occurrence of *BRAF* mutations in melanoma, BRAF inhibitors, vermurafenib and dabrafenib, have been developed. These two inhibitors are currently being used to treat melanoma patients with BRAF^V600E^ mutations [16]. However, these inhibitors are less effective in the case of other rarer *BRAF* V600 mutations such as *p*.V600D. Furthermore, it has been demonstrated that melanomas develop resistance to these inhibitors due to secondary mutations in V-Ras neuroblastoma RAS viral oncogene homolog (*NRAS*) and other mutational alterations [17,18]. Hence, a combined therapy which involves BRAF and mitogen-activated protein kinase kinase 1 (MAP2K1/MEK) inhibitors may be used to improve patient response in cases where the tumour contains multiple targetable mutations [19].

NRAS is a member of RAS signalling proteins. RAS proteins are located in cell membranes, and they switch to the active GTP-bound state from their inactive GDP-bound state when they interact with their respective receptors. In their active GTP-bound state, they recruit RAF proteins to the cell membrane. RAF proteins are activated by phosphorylation, and they further phosphorylate downstream proteins in the MAPK pathway [20]. *NRAS* mutations are the second most frequent somatic mutations in human melanomas with a 15–38% frequency of occurrence [21]. Most *NRAS* mutations occur at codon 61 in exon 3, where glutamine is mutated to either arginine or lysine [22]. This mutation causes the constitutive activation of RAS protein, resulting in malignant cell growth through the constitutive activation of MAPK and phosphatidyl inositol 3- kinase (PI3K) pathways [23].

Neurofibromatosis type 1 (*NF1*) is a tumour suppressor gene located at 17q11.2 [24]. It encodes neurofibromin protein which is implicated in the downregulation of RAS proteins. The loss of *NF1* results in prolonged activation of RAS proteins, which ultimately leads to uncontrolled cellular proliferation [25]. Homozygous deletion of *NF1* was identified in melanoma cell lines. In addition, the absence of neurofibromin has been reported in primary melanomas, which led to the proposal that *NF1* may function as a tumor suppressor gene and is crucial in the progression of melanoma. Exome sequencing studies demonstrated *NF1* mutations in 12–30% of melanoma [26]. Interestingly, 20–30% of *NF1* deletions have been found in *BRAF* and *NRAS* wild type melanomas suggesting that *NF1* may be implicated in MAPK pathway activation [25].

The Rac family small GTPase1 gene (*RAC1*) encodes a GTPase, which is a part of the RAS superfamily of small GTP-binding proteins. Whole exome sequencing studies have identified recurrent mutations in *RAC1*, particularly involving proline to serine mutation at position 29 [27]. Functional analysis of wild type and mutant *RAC1* expression in transiently transfected mouse melanocytes demonstrated that the expression of mutant *RAC1*, but not wild type *RAC1*, enhanced the phosphorylation of extracellular signal-regulated kinases (ERK), cell proliferation and cell migration. This finding confirmed the role of *RAC1* mutation in the activation of MAPK downstream signalling in melanocytes [25,28].

The phosphatase and tensin homolog gene (*PTEN*) is a tumor suppressor gene located in chromosomal region 10q23.31. Deletion of *PTEN* is well-known in many cancer types [29]. *PTEN* aberrations have been reported in 28–43% of melanomas [30], and allelic mutations and deletions of *PTEN* have been reported in melanoma cell lines [30,31]. Overexpression of *PTEN* in melanoma cell lines leads to inhibition of cell growth, indicating a tumour suppressive role for *PTEN* [31]. Loss of *PTEN* is implicated in activation of the PI3K signalling pathway, and has been shown to promote cell survival suggesting that it has a crucial role in melanoma progression [30,32].

The V-Kit Hardy-Zuckerman 4 feline sarcoma viral oncogene homolog gene (*c-KIT*) encodes a type III receptor tyrosine kinase and acts as a receptor for stem cell factor (SCF). The c-KIT-SCF signalling pathway is crucial for melanocyte development, survival, proliferation, differentiation, and migration [33]. *c-KIT* mutations in exon 11, 13, 17 and 18 have been found in a very small subset of malignant melanomas, with lysine 642 to glutamine (K642E) and leucine 576 to proline (L576P) being the most common mutations. Functional analysis of a *c-Kit* mutant mouse cell line, Melan-a, demonstrated increase in the activation of the PI3K pathway and activation of the MAPK pathway in hypoxic conditions [34].

The melanocyte-inducing transcription factor (*MITF*) is also mutated in some sporadic melanomas [35]. A genome-wide study utilizing single-nucleotide polymorphism (SNP) arrays demonstrated that there was substantial amplification of *MITF* in 10% of primary cutaneous melanomas and in 21% metastatic melanomas. These observations indicated that *MITF* is an oncogene in melanoma [36].

The guanine nucleotide binding protein subunit alpha Q (*GNAQ*) and guanine nucleotide binding protein subunit 11 genes (*GNA11*) contain oncogenic mutations that are found at a frequency of 30–40% in uveal melanomas [37,38].

Other lower frequency mutations in melanoma include rare mutations in V-Akt murine thymoma viral oncogene homologs 1 and 3, (*AKT1* and *AKT3*), and mutations in *MAP2K1* and *MAP2K2*, which have demonstrated constitutive activation of the ERK pathway [39]. However, the functional relevance of many of these mutations is unclear at present [40]. In addition, inactivating mutations in *MAP3K5* and *MAP3K9* have been discovered [41]. Exome sequencing studies carried out in 14 matched normal and metastatic samples identified that 33% contained mutations in glutamate ionotropic receptor NMDA type subunit 2A (*GRIN2A*) and 3% contained recurrent mutations in transformation/transcription domain associated protein (*TRRAP*) [42]. Whole genome sequencing analysis of 25 metastatic melanoma samples matched with germline DNA identified phosphatidylinositol-3,4,5-trisphosphate dependent Rac exchange factor 2 (*PREX2*) as a significantly mutated gene with mutation frequency of 14% in an extended study of 107 human melanomas. The overexpression of *PREX2* in vivo initiated the expression of variant proteins, which were involved in oncogenic activity in melanoma cells [43].

### 2.2. The Mutational Landscape of Familial Melanomas

Familial mutations also cause malignant melanomas. These mutations increase the risk of acquiring malignant melanoma by about two-fold [44]. Cyclin-dependent kinase 2a (*CDKN2A*) was the first high-risk susceptibility gene identified in melanoma. This gene is located on chromosome 9p21 and encodes two proteins, p16^INK4A^ and p14^ARF^ which are associated with the cell cycle and apoptosis regulation. p16^INK4A^ interacts with cyclin dependent kinase 4 (*CDK4*) to prevent it from phosphorylating the retinoblastoma protein (RB), which leads to cell cycle arrest in the G1 phase [45]. On the other hand, p14^ARF^ binds to human double minute-2 (HDM2) protein to induce degradation of HDM2, which is responsible for ubiquitination of tumour protein p53 (p53). This results in the stabilization of p53. Hence, the loss of p16^INK4A^ induces transition from G1 to S phase, and re-entry into the cell cycle, while the loss of p14^ARF^ results in destabilization of p53, which in turn initiates cellular growth [44]. Recurrent germline mutations in the *CDK4* gene have been reported in melanoma. *CDK4* is located in 12q13.6 and encodes a protein that interacts with p16^INK4A^. The germline mutations in *CDK4* disrupt its interaction with p16^INK4A^, leading to cell cycle progression [46]. Recent findings have also identified germline mutations in the promoter region of telomerase reverse transcriptase (*TERT*), the gene that encodes the catalytic subunit of telomerase. *TERT* mutations have also been found as a somatic alteration at a high rate in melanoma. The mutations in *TERT* lead to the binding of E-twenty-six 1 (ETS1) transcription factors to the *TERT* promoter, which consequently results in fourfold increased transcriptional activity of *TERT* [47]. Furthermore, it has been demonstrated that there is a close association between the *TERT* expression and activation of the MAPK pathway through ETS1 [48]. 

GWAS studies in melanoma have identified common intermediate and low-risk melanoma susceptibility genes. The *MITF* gene, located at chromosomal region 3p14, was identified as an intermediate-risk melanoma susceptibility gene after a germline mutation was identified that resulted in a glutamic acid to lysine change at codon 318 (p.E318K) [49]. This mutation was associated with melanoma and renal cancer.

More than 15 low-risk melanoma susceptibility genes have been identified by GWAS studies including melanocortin type 1 receptor (*MC1R*), solute carrier family 45 member 2 (*SLC45A2*), oculocutaneous albinism II (*OCA2*), agouti signalling ptotein (*ASIP*), tyrosinase (*TYR*), tyrosinase related protein 1 (*TYRP1*) [50]. *MC1R* is the best characterized low risk susceptibility gene. It is involved in normal pigment variation in humans, and it encodes a G-protein coupled receptor for α-melanocyte stimulating hormone (α-MSH). Binding of α-MSH receptor to MC1R in the normal situation, results in activation of adenylate cyclase, which consequently results in an increase in cyclic adenosine monophosphate (cAMP), leading to a switch in melanin production from pheomelanin pigments to eumelanin [51]. Five variants, p.D84E, p.R142H, p.R151C, p.R160W and p.D294H have been defined as red hair colour (RHC) variants, and are associated with extensive freckling, fair skin colour and poor tanning ability. Other variants are termed as non- RHC variants and have a weaker or no association with red hair colour. Several studies have shown increased risk of melanoma among the RHC variants of *MCIR* [52]. Factors such as UV exposure, allele frequencies and genetic background may contribute to the variation in melanoma risk associated with *MC1R* [53].

### 2.3. Mutational Landscape Studies of Metastatic Melanomas

Incremental disruption of oncogenic pathways has been observed as an important mutational contribution to melanoma progression [54]. In particular, somatic alterations in PI3K and p53 pathway genes were shown to be involved in the progression of melanoma to thick primary lesions [54]. In this regard, somatic mutation and clonal selection, eventually leading to multiple subclones and extensive heterogeneity within the tumour, is a key principle of the clonal evolution model of metastasis (see Section 4.1). However, no mutations have been specifically associated with metastatic melanoma [54]. Mutations do nevertheless contribute to the tumour mutation burden (TMB), and this together with tumour neoantigens, is associated with improved response to immune checkpoint inhibitor treatment [55,56]. Transcriptional changes are frequently observed in metastatic melanomas, which will be covered in more detail in the following sections.

## 3. Gene Expression Alterations and Cell Signalling Pathways Underlying Melanoma Initiation and Progression to Invasion and Metastasis

**Figure 1:**
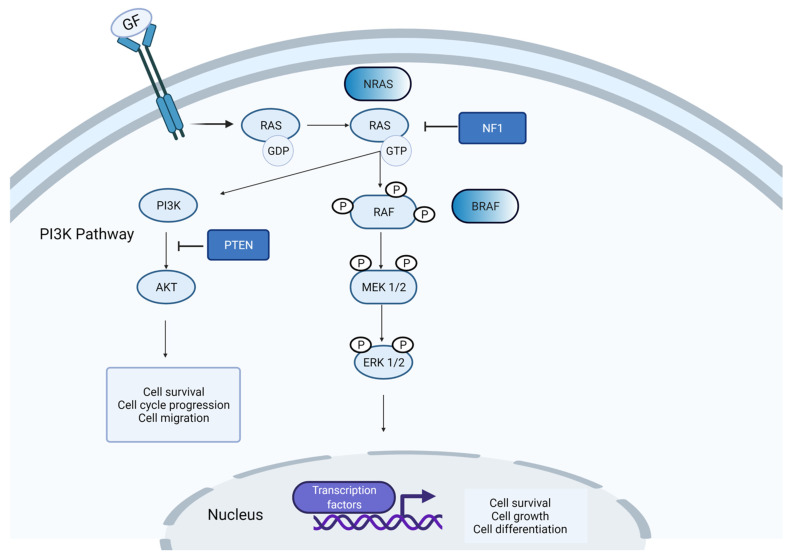
Schematic of cellular events in the MAPK pathway. MAPK signalling can be initiated by receptor tyrosine kinases (RTKs). Once activated, the RTKs stimulate downstream signalling by RAS, RAF and MEK and ERK, finally resulting in activation of several transcription factors in the nucleus. RAS-GDP is also involved in PI3K signalling through AKT activation.

Most of the mutations discussed above affect proteins involved in major signalling pathways in melanoma. The RAS/RAF/MEK/ERK pathway, also known as MAPK pathway, is the main regulatory pathway implicated in melanoma. This pathway is involved in cell growth, migration, differentiation, proliferation and apoptosis [57]. The MAPK pathway can be initiated by either binding of receptor tyrosine kinases (RTKs) or integrin adhesion between the cell membrane and the extracellular matrix (ECM) (Figure 1). This binding activates RAS proteins by converting them from a GDP-bound state to a GTP-bound state. In its active state, RAS initiates the phosphorylation of RAF proteins. The activated form of RAF further initiates the downstream signalling by phosphorylation of MEK and consequently ERK. Activated ERK is translocated to the nucleus, where it regulates the expression of several transcription factors, which are associated with cell-cycle progression and differentiation [58]. Mutations in BRAF result in constitutive activation of MEK and ERK. Mutations in NRAS result in deactivation of GTP from the RAS complex and constitutive activation of downstream signalling [59]. As already mentioned, deletions involving the *NF1* tumor suppressor gene lead to uncontrolled cell proliferation and invasion [60].

Another signalling pathway involved in melanoma is the PI3K pathway, which is activated by the binding of insulin-like growth factor 1 (IGF-1) to insulin-like growth factor receptor 1 (IGFR-1) [61]. Activation of PI3K results in increased phosphatidylinositol phosphate (PIP3) production, which then functions as a docking site for 3-phosphoinositide-dependent protein kinase 1 (PDK1). PDK1 activates protein kinase B (AKT), encoded by the *AKT1* gene, through phosphorylation. The activated form of AKT regulates several proteins associated with cell cycle progression, survival and migration (Figure 1). *PTEN* inactivates the downstream targets of the PI3K pathway [44]. Therefore, it has an important role in inhibiting cell survival, cell growth and proliferation during oncogenic transformation.

**Figure 2:**
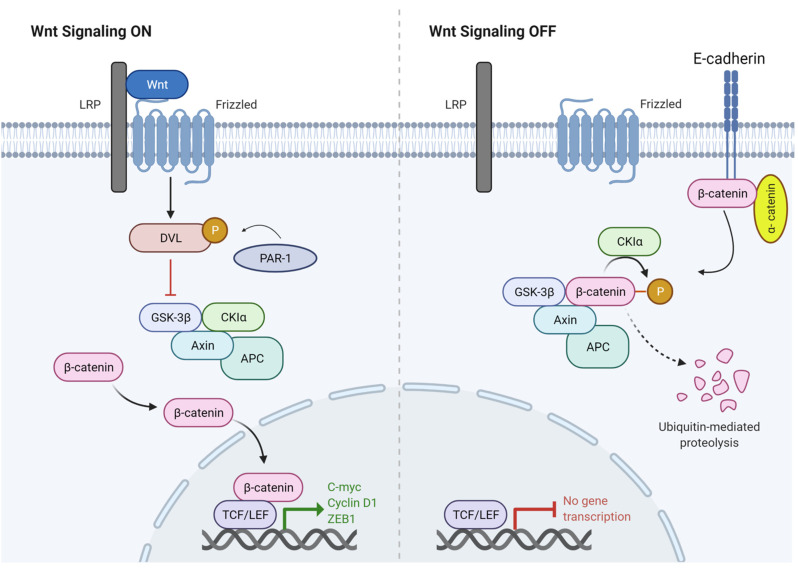
The canonical Wnt signalling pathway. Wnt binding causes inhibition of the GSK3-CK1α-APC-Axin complex through dishevelled protein (DVL). DVL gets activated by phosphorylation through PAR-1. As a result of the inhibition activity, free β-catenin accumulates in the cytoplasm and then translocates to the nucleus to mediate the transcriptional activation of oncogenic target genes such as cyclin D1, C-MYC and ZEB1. In absence of Wnt ligands, β-catenin is bound to E-cadherin as a part of adherens junction complex. The β-catenin destruction complex composed of Axin, GSK3, CK1α and APC causes phosphorylation of β-catenin through GSK3 and CK1α leading to proteasomal degradation of β-catenin.

Wingless type MMTV integration site (Wnt) signalling controls normal tissue function, embryonic development, proliferation and migration [62]. The canonical Wnt pathway is activated by interaction of Wnt with liproprotein receptor-related protein (LRP-5/6) receptors and frizzled family receptor proteins (FZD). The binding of Wnt to LRP/FZD complex leads to the release of β-catenin from E-cadherin, causing inhibition of the Axin-APC-GSK-3β-CK1α complex through dishevelled (DVL) which is phosphorylated by proteinase-activated receptor 1 (PAR-1). Furthermore, the inhibition of glycogen synthase kinase 3 beta (GSK-3β) results in accumulation of β-catenin the cytoplasm. Cytoplasmic β-catenin migrates to the nucleus and mediates transcription of Wnt target genes, v-myc avian myelocytomatosis viral oncogene homolog (*c-MYC*), Cyclin D1 (*CCND1*), zinc finger E-box binding homeobox 1 (*ZEB1*), by interacting with its binding partner T cell factor/lymphoid enhancer factor (TCF/LEF) family of transcription factors. On the other hand, when Wnt is inactive, β-catenin is inactivated by phosphorylation through GSK-3β and casein kinase 1 alpha 1 (CK1α) which are a part of destruction complex formed by Axin, adnomatosis polyposis coli protein (APC), GSK-3β, and CK1α. Subsequently, β-catenin goes through ubiquitin mediated degradation [62] (Figure 2).

Normally, Wnt signalling is implicated in the development of melanocytes from neural crest precursors [63], whereas abnormal activation of Wnt pathway is associated with melanoma development. Overexpression of *WNT5A* is known to activate the Wnt pathway through its interaction with FZD which consequently leads to an increase in β-catenin signalling, and increases cell motility and invasion to promote metastasis [64].

Other G-protein coupled receptors (GPCRs), apart from FZD, such as MC1R, endothelin receptor (EDNR), C-X-C motif chemokine receptor (CXCR) and PAR1 are also implicated in metastatic melanoma progression [65,66,67,68].

**Figure 3:**
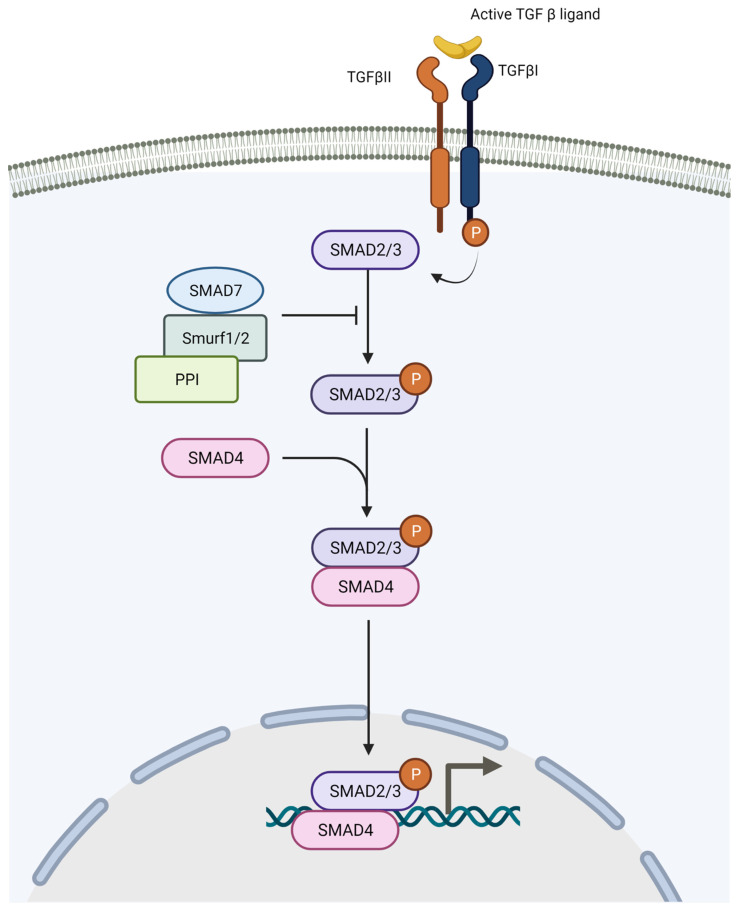
The TGFβ signalling pathway. Upon binding of the TGFβ ligand to the TGFβ type II receptor, the latter phosphorylates the TGFβ type I receptor which further phosphorylates SMAD2/SMAD3. Activated SMAD2/3 binds to SMAD4 and accumulates in the nucleus to regulate the transcription of target genes. SMAD7 acts as an inhibitor of TGFβ signalling. It recruits phosphatase PP1 or ubiquitin ligases Smurf1, Smurf2, to induce dephosphorylation or proteasomal degradation of receptor complexes.

Transforming growth factor β (TGFβ) is known to downregulate E-cadherin and upregulate N-Cadherin expression by initiating the transcription of snail family transcription repressor 1 (*SNAI1*), SLUG (*SNAI2*) and *ZEB1*. TGFβ is initiated by the binding of TGFβ ligand to the TGFβ type II receptor, which upon binding phosphorylates the TGFβ type I receptor (TβRI). Phosphorylated TβRI further phosphorylates mothers against decapentaplegic homolog 2 (SMAD2) and SMAD3 proteins. The phosphorylated SMAD2/3 proteins interact with SMAD4 protein to form heterocomplexes that act as transcription factors and induce the transcription of target genes. SMAD7 is another member of the SMAD proteins, which acts as an inhibitor of TGFβ signalling by ubiquitinating SMAD2/3 proteins through its direct binding to the receptor complex of the TGFβ receptor (Figure 3). Several findings have demonstrated there is high expression of TGFβ in melanoma cell lines [69,70].

## 4. Intra-Tumoral Heterogeneity in Melanoma as A Precursor to Metastasis

Cancer is a highly heterogenous disease. The following models have been proposed and are the most popular models to explain heterogeneity within tumours.

### 4.1. The Clonal Evolution Model

**Figure 4:**
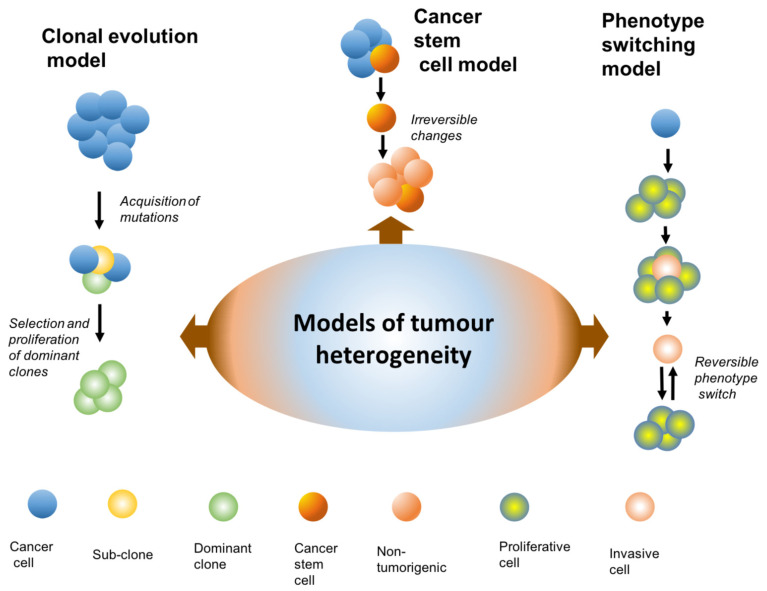
Models of the tumour heterogeneity. The clonal evolution was the first model. In this model the cancer cells evolve progressively due to genetic changes. The genetic instability produces many variants. Most variants die due to metabolic or immunologic disadvantage. The variants that have a selective advantage become the dominant sub-population until an even more favourable variant appears. Over time there is sequential selection of sub clones through evolutionary selection. The cancer stem cell model proposes that tumour heterogeneity arises because cancer cells give rise to stem cell-like cells which have a capacity to self-renew and differentiate into multiple cell types, just like the normal stem cells. The daughter cells of cancer stem cells are unidirectional and are not able to de-differentiate back into cancer stem cells. Hence, this model does not explain the plasticity represented by cancer cell subpopulations. The phenotype switching model is the most recently proposed model. This model shows a bidirectional relationship between cancer cell sub-populations. For details refer to the Figure 5.

The clonal evolution model proposed by Peter C. Nowell in 1976 states that tumour cell initiation occurs through genetic changes in a single cell which make it “neoplastic” and provide it with a selective growth advantage. Over time, the mutant cells proliferate. A cell with a selective growth advantage becomes a precursor to the subsequent mutant cell subpopulations [71]. This model fits well with the hierarchy of clinical stages of melanoma from nevi and primary melanoma, which are relatively less aggressive, compared to the aggressive metastatic stages. However, this model has several deficiencies, including failure to explain the lack of mutations that have been identified specifically associated with metastatic melanoma (Figure 4).

### 4.2. The Cancer Stem Cell Model

The cancer stem model posits that cancer progression occurs through a small number of cells within the cancer, called cancer stem cells. Such cells, like normal stem cells, are able generate subpopulations of different phenotype within the cancer and are able to self-renew to maintain the tumour [72,73]. This model has been widely accepted in leukaemia, brain tumours, carcinomas, breast cancers and colon cancer [73,74,75,76,77,78,79]. Several markers have been identified in various cancers that are able to distinguish between cancer stem cells and non-tumorigenic cells [9,80]. However, some studies have shown that stem cell markers may also be expressed in the non-tumorigenic cells along with the cancer stem cells and so the validity of this model has been questioned in many cancers including melanoma [81,82,83].

According to the cancer stem cell model (CSC model), cells with tumorigenic properties give rise to non-tumorigenic cells in a hierarchical manner, similar to normal stem cell differentiation. Additionally, the cancers that follow this model have shown that daughter cells that derive from the precursor cancer stem cells have rare, or no ability to form tumours. Hence, the CSC model follows an irreversible pathway of genetic changes in driving heterogeneity in the cancer cells (Figure 4). [73,74,75,77,79]. It has been shown that melanoma cells can exhibit stem-like characteristics because they show phenotypic heterogeneity in vitro and in vivo [84], and they express genes that are normally expressed during embryonic development [85]. In addition, they can differentiate into multiple lineages including mesenchymal, endothelial and neural [86,87,88]. However, evolving studies that have been carried out on melanoma and other cancers, have demonstrated that there is plasticity between non-tumorigenic and tumorigenic cancer cells, suggesting that the phenotypic changes are bidirectional in nature, as compared to the unidirectional nature of the CSC model [89,90,91,92,93,94,95]. This suggests more compatibility with the phenotype switching model (Figure 4). In addition, it has been shown that essentially all melanoma cells, and not just a small subset of stem-like cells present in a melanoma tumour are able to form a tumour when implanted into immunodeficient mice [81,96]. These studies suggest that the CSC model is not entirely applicable to melanoma progression.

### 4.3. The Phenotype Switching Model

**Figure 5:**
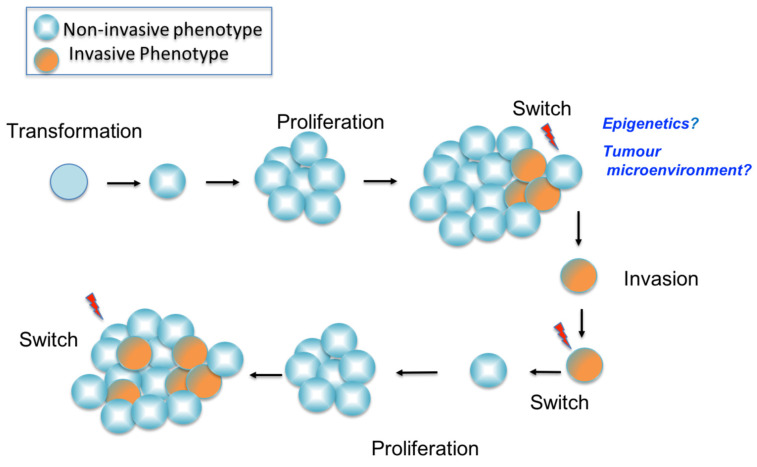
Phenotype switching in melanoma. In the early phase melanoma cells show a non-invasive phenotype with proliferative characteristics. Following this a hypothetical switch, brought about by the factors in the tumour microenvironment such as hypoxia or inflammation, or perhaps by epigenetic changes, gives rise to a phenotype with a high invasive capacity. The invasive cells escape the primary site and upon reaching a suitable distant site, revert to the non-invasive phenotype and the cycle is repeated. Each switch in phenotype is accompanied by the expression of a gene signature specific to each phenotype.

The phenotype switching model for melanoma was proposed by Hoek et al., in 2008 [97]. This model proposes that melanoma progression is not just the result of genetic mutations, but that it relies greatly on gene transcription. In particular, the ability of melanoma cells to invade is suggested to depend on phenotypic plasticity, which drives melanoma invasion. Moreover, in transcriptomic studies, melanomas have been shown to demonstrate distinct phenotypes, with distinct gene expression signatures, that are reversible during metastatic progression [97,98,99,100] (Figure 5).

## 5. Single Cell Sequencing and Spatial Transcriptomics: A Step towards Understanding Tumour Complexity, Tumour Progression and Drug Resistance

Tumours are complex tissues with various cell types including cancer cells, stromal cells and immune cells. Treatment response in a tumour can vary depending on the interaction between different types of cells within the tumour, tumour microenvironment and immune responses. Given the heterogeneous nature of tumours, the conventional methods of analysing the ‘bulk’ tumour tissue do not give complete information about the tumour. To this end, single cell sequencing in tumours has helped greatly in determining different cell types and their expression markers. This information can ultimately help in predicting the patient’s response to drugs and overall outcome. Recently, it has been shown that spatial information is also useful in addition to single cell analysis, because the spatial arrangement of cells in a tumour affects the tumour microenvironment [101].

A study carried out by Tirosh et al., [102] provided an in-depth analysis of cell types within melanoma tumours. The study identified variable cell cycle markers characterised by highly proliferative cancer cells and slow cycling cells which expressed genes involved in drug resistance. This profile was similar to gene expression profiles characterised in non-invasive and invasive phenotypes in melanoma in previous studies [97,103]. In addition, the study also identified that the activity of T cells varied, and was different based on the spatial context of the cells [102].

Another study by Thrane et al., [104] demonstrated that single cell genomics together with spatial transcriptomics could help greatly in studying transcriptional heterogeneity in stage III melanoma in relation to tumour microenvironment. Single cell sequencing in conjunction with spatial transcriptomics enabled understanding of the biological implications of therapy response, depending on the tumour microenvironment [104]. In future, these two technologies may better define the different cell types within a tumour, with implications for therapy response and outcome.

## 6. Transcriptomic or Epigenomic Differences between Invasive and Non-Invasive Phenotypes in Melanoma Cell Lines

Phenotype switching, in many respects, is analogous to epithelial to mesenchymal transition, while incorporating simultaneously its reverse process, mesenchymal to epithelial transition. A significant body of work has already investigated transcriptomic and epigenomic differences between invasive and non-invasive melanoma phenotypes, as described in the following sections.

### 6.1. Transcriptomic Differences between Invasive and Non-Invasive Phenotypes

Hoek et al., in 2008 carried-out microarray analysis of 86 metastatic melanoma tissue samples that belonged to three different cohorts [97]. This gene expression analysis identified two distinct melanoma cell phenotypes based on distinct transcriptional profile of 105 genes—proliferative and invasive. The proliferative group, as the name suggests, demonstrated high proliferative capacity, but a weak invasive capacity, and this group was characterized by high expression of genes associated with neural crest differentiation and cell cycle control such as *MITF* and its target genes, *TYR*, dopachrome tautomerase (*DCT*), melan-A (*MLANA*). The invasive group demonstrated an invasive phenotype and was characterized by down regulation of the genes involved in the proliferative group, and upregulation of *WNT5A,* POU domain class 3 transcription factor 2 *(POU3F2*)*,* and AXL receptor tyrosine kinase *(AXL*) and genes involved in the interaction with extracellular environment, such as lysyl oxidase (*LOX*), *COL5A1* and thrombospondin 1 (*THBS1*). An intermediate group was also identified in this study which suggested that the two main subtypes also have a transient intermediary group (Cohort B).

These gene expression differences were ascribed to invasive and proliferative states, and the phenotype switching model was further validated in vivo. Proliferative and invasive cells were both found to initiate tumour growth in mice models, indicating that the two phenotypes were not static and could switch back and forth from one phenotype to the other [97].

Several publications have described a panel of melanoma cell lines (NZM cell lines), derived from metastatic melanomas of New Zealand melanoma patients [103,105,106,107,108,109,110,111,112,113,114,115,116,117]. Many of these melanoma cell lines, which are derived from metastatic melanomas, are non-invasive, while others are invasive [103,108], and taken together, the NZM cell line panel has been shown to exhibit a diverse range of phenotypes. Extensive molecular characterization of the NZM lines has revealed that the panel closely matches the spectrum of molecular alterations observed in melanomas analyzed from patients in the general population [110]. An analysis of the invasiveness of NZM melanoma cell lines indicated that, as has been observed in other melanoma cell line panels [97,98,118,119], the NZM cell lines could be grouped into both invasive and non-invasive subgroups, with a characteristic gene expression signature corresponding to each subgroup [103,108]. Thus, for these two sub-groups of the NZM cell lines, the reported patterns of gene expression were similar to those of Hoek et al., 2008 [97,103,108].

In particular, the non-invasive subgroup was characterized by high expression of developmental and lineage specific genes such as *MITF*, *EDNRB*, *DCT* and *TYR* whereas the invasive group showed high expression of genes involved in interaction with the extracellular environment such as hypoxia inducible factor 1 subunit alpha (*HIF1A*), versican (*VCAN*) and plasminogen activator urokinase receptor (*PLAUR*). These results were further validated using migration assays following *MITF* knockdown [103]. The knockdown of *MITF* in the non-invasive cell lines increased the migration capacity by approximately 4-fold, suggesting that *MITF* may be a master regulator of the phenotypes and that the two phenotypes may be reversible. Additionally, validation with publicly available melanoma patient datasets demonstrated that the gene expression signature overlapped with both primary and metastatic tumours, suggesting that invasive potential in melanoma is independent of tumour stage [103].

Further studies have demonstrated that the heterogeneous nature of melanoma is in part due to the phenotypic plasticity of the melanoma subtypes and that these phenotypes occur independently of the mutational status or tumour stage [120,121]. However, while invasiveness is an important characteristic of metastatic melanomas, a full description of the mechanisms by which melanoma becomes invasive remain unclear, but these findings suggest that factors other than mutations are involved in this mechanism. Furthermore, it has been proposed that tumour microenvironmental changes such as hypoxia, nutrient deprivation and inflammation could initiate the transition between these two phenotypes [122,123,124,125]. For example, hypoxia drives HIF1α-dependent phenotype switching [126].

A number of signalling pathways have been identified that regulate phenotype switching, and which potentially explain the resistance of metastatic melanoma to therapy [127]. In this regard, several intermediate melanoma states (between invasive and non-invasive) have been observed, which have been shown to exhibit increasing drug resistance (reviewed in [127]). The variable response to melanoma therapies could be due to the notion that phenotype switching generates different sub-populations of cells in response to the changing tumour microenvironment. A better understanding of the underlying mechanism of phenotype switching and of the regulation of the different phenotypes will be helpful to take a better approach in designing therapies to target resistant melanoma cells.

### 6.2. Epigenomic Differences between Invasive and Non-Invasive Phenotypes

Mounting evidence suggests that mutational status is insufficient to predict cancer metastasis. Further, mutations are also insufficient to explain phenotypic plasticity in melanoma, including phenotype switching. Therefore, investigators have begun to employ epigenetic and epigenomic studies to explain phenotype plasticity and phenotype switching in melanoma [128,129,130,131,132,133,134,135,136,137]. These studies include chromatin remodeling, as well as genome-wide DNA methylation analysis, together with integrated transcriptomic analysis of protein-coding regions to investigate mechanisms that regulate phenotype switching between the invasive and non-invasive subtypes of cutaneous melanoma [108,133,138,139]. Epigenetic and epigenomic studies could help to explain why melanoma cells present with stem-like characteristics in association with phenotypic heterogeneity, both in vitro and in vivo [84], and why melanomas often express genes that are normally expressed during the embryonic development [85], and are able to differentiate into multiple lineages including mesenchymal, endothelial and neural [86,87,88]. Furthermore epigenomic changes could help to explain plasticity between non-tumorigenic and tumorigenic cancer cells, whereby the epigenomic changes could be bidirectional in nature, supporting the phenotype-switching model [97] as compared to changes that are unidirectional in nature in the CSC model. For example, transcription factors, such as MITF in melanoma have been proposed to drive dedifferentiation and invasion through epigenetic regulators, like the polycomb repressive complex 2 protein, enhancer of zeste homolog 2 (EZH2) [134,140]. In addition, it is worth noting that mutations also occur in the *EZH2* gene in approximately 3% of melanomas [141], and furthermore that EZH2 has been shown to cooperate with DNA methylation changes to down-regulate key tumour suppressors and interferon gene signatures [142]. Among the tumour suppressor genes with frequent promoter hypermethylation in melanomas, causing repression of transcription, are *CDKN2A* [143,144], Ras association domain family member 1 (*RASSF1A*) [145], and *PTEN* [146,147], with the latter two particularly becoming hypermethylated in later stages of the disease. Epigenetic changes and their prevalent role in malignant melanoma were recently reviewed [138].

In a recently published study, Motwani et al., [108] carried out a transcriptomic analysis in the NZM melanoma cell lines, where over fifty percent of differentially expressed genes (DEGs) identified in their study overlapped with DEGs identified previously in other studies associated with invasive and non-invasive phenotypes [98,103,139], including EMT transcription factors as such as *ZEB1* and *SNAI1* [106,148]. Moreover, twelve of the DEGs (i.e., collagen genes (*COL5A1*, *COL1A2*, *COL6A2*, *COL11A1*), thrombospondin 2 *(THBS2*), cysteine rich secretory protein LCCL domain containing 2 (*CRISPLD2*)*,* procollagen C-endopeptidase enhancer *(PCOLCE*)*,* inhibin subunit beta A *(INHBA*)*, VCAN,* lumican (*LUM*)*,* platelet-derived growth factor receptor beta (*PDGFRB*)*,* and thy1 cell surface antigen (*THY1*)) were among the top upregulated and downregulated DEGs matching with a sixty four-gene multi-cancer invasion signature that was associated with common epithelial cancers including ovarian cancer, colon cancer, breast cancer, gastric cancer and lung cancer, and also some non-epithelial cancers such as neuroblastoma and Ewing’s sarcoma [149,150]. Intriguingly, peroxidasin (*PXDN*), a fibroblast protein involved in the formation of extracellular matrix [151], was observed to have the highest relative expression in the invasive group. Peroxidasin has been suggested to have a crucial role in melanoma invasion in vitro and in vivo [152]. In addition, DEGs included several hypoxia signature genes that were previously reported in breast cancer [153].

In addition to transcriptomic analysis, Motwani et al., [108] carried out an integrated genome-wide DNA methylation analysis, and observed forty-nine differentially methylated fragments (DMFs) between the invasive and non-invasive cell lines. Of these, DMFs associated with arginine vasopressin induced 1 (*AVPI1*), high mobility group 20B (*HMG20B*), synaptojanin 2 (*SYNJ2*), and B cell lymphoma 3 transcription coactivator (*BCL3*) overlapped with the DEGs. Most of these DMFs occur near to genes associated with cancer, and in sequences that have gene regulatory functions, such as transcription factor binding sites, enhancer regions, histone marks and DNAse I hypersensitivity sites. For example, *SYNJ2,* which encodes the 5’-inositol lipid phosphatase, synaptojanin 2, has been reported to promote cell migration and invasion in breast cancer and is associated with a high risk of colorectal cancer [154,155]. The DMF associated with *SYNJ2* overlapped with CCCTC binding factor (CTCF) transcription factor binding sites. In another example, high expression of the *BCL3* transcriptional co-activator is correlated with poor prognosis in colorectal cancer and gliomas [156,157]. DMFs identified in *HMG20B* overlapped with a CTCF binding site, and another contained in an intron region has previously been reported as an epigenetic factor involved in chromatin organization [158], being required for cell division and cytokinesis in association with the breast cancer type 2 susceptibility protein (BRCA2) [159]. Hypomethylation of *HMG20B* was positively correlated with gene expression. Additionally this DMF region overlapped with regions involved in epigenetic regulation, including through the histone deacetylase 2, HDAC2, which is an epigenetic repressor involved in regulating transcription and cell cycle progression [160]. It also overlapped with epigenetic modifier lysine demethylase 1A (KDM1A), essential for cell growth and interacts with promoters or enhancer regions [161], and chromodomain helicase DNA binding protein 4 (CHD4), which is also known to be involved in epigenetic repression. The *AVPI1* gene promoter contained several DMFs that were hypermethylated with low expression of *AVPI1* in invasive cell lines. *AVPI1* knockdown has been reported to significantly inhibit the induction of cell death by MLN4924, an inhibitor of neural precursor cell expressed developmentally down-regulated 8 (NEDD8)-activating enzyme, and which is involved in cancer progression [162]. Furthermore, high levels of *AVPI1* expression have been identified in association with cell cycle entry [163], and also to be involved in activating the MAPK pathway [164].

The total number of protein-coding genes (*n* = 4) identified to have an overlap of expression and methylation difference (i.e., having both a significant gene expression and significant DNA methylation difference) in Motwani et al.,’s integrated transcriptomic and DNA methylome analysis seems to be a relatively small number at face value [108]. Several reasons come to mind as to why more overlap was not observed; (1) much more in-depth analysis may be helpful; (2) individual cells in culture, or in a bulk tumour, might be dynamically and constantly moving between, and ‘sampling’ multiple different phenotypic states, and therefore carrying out single cell analysis could provide a much greater resolution study to identify further genes that overlap epigenetically and transcriptionally; (3) the majority of the changes in transcription and methylation could involve the “dark matter” of the genome, including the intergenic regions, such as transcribed enhancer elements, which are associated predominantly with non-coding transcripts, and this, together with demethylation events, could centre on repetitive elements, including transposable elements. Indeed, quite likely, all three of the above possibilities (and more) would account for the results seen. Therefore, if one attempts to visualize the trajectory of a tumour cell in Waddington’s landscape model [165], then a relatively simple “rheostat” notion of phenotype switching could be just one well-worn pathway (i.e., ‘valley‘) among many side valleys that a tumour cell could also hide in. This could have implications for understanding pathways of metastasis and for drug-resistance in melanoma.

## 7. Conclusions

The literature shows that metastatic melanoma is a complex and highly heterogeneous type of skin cancer [81]. To date, the published literature has found little evidence to support the notion that there is a mutational mechanism leading to melanoma metastasis. In contrast, accumulating evidence supports the notion that transcriptomic differences contribute to the phenotypic difference observed between invasive and non-invasive melanoma cell phenotypes. In this regard, expression profiling of melanoma cell lines derived from metastatic melanoma patients has revealed at least two, and possibly four or more gene expression signatures, corresponding to different transcriptomic states of melanoma, occur with different gene regulatory pathways [120], which may help to distinguish between invasive, non-invasive, and other melanoma cell phenotypes. It has been suggested that melanoma cells undergo adaptations, including (but possibly not limited to) phenotypic switching, in order to adopt alternative metabolic states, due at least in part to changes in the tumour microenvironment, including hypoxic, nutrient deficient, toxic, or immune hostile conditions [97]. In line with this, melanoma cells may often co-exist with, and reversibly switch between subpopulations harbouring transcriptional signatures of melanocytic differentiation and therapeutic sensitivity, and other subpopulations of invasive, dedifferentiated and therapy-resistant melanoma cells in bulk tumours. It is possible that bulk tumours are able to generally incorporate aspects of at least two of the three models simultaneously in the biology of their various tumour cell subpopulations. Together with transcriptomic differences, it has also been proposed that epigenetic differences could be associated with phenotype switching, tumour cell evolution, acquisition of invasiveness, and similar adaptations. A study to investigate genome-wide DNA methylation profiling associated with invasive melanoma cell behaviour, as a relatively stable epigenomic alteration that frequently involves protein-coding genes, revealed multiple such changes, including changes overlapping with four differentially expressed protein-coding genes [108]. The potential role of genomic alterations in invasion and metastasis in melanoma still requires considerable further work, although initial studies suggesting that genomic and epigenomic alterations, which involve phenotypic changes in melanoma cells (including phenotype switching, and similar dedifferentiation-associated changes), are beginning to appear.

## Data Availability

Not applicable.
